# Option generation in decision making: ideation beyond memory retrieval

**DOI:** 10.3389/fpsyg.2014.01584

**Published:** 2015-01-22

**Authors:** Fabio Del Missier, Mimì Visentini, Timo Mäntylä

**Affiliations:** ^1^Department of Life Sciences, University of TriesteTrieste, Italy; ^2^Department of Psychology, Stockholm UniversityStockholm, Sweden

**Keywords:** option generation, decision structuring, decision making, memory, ideation

## Abstract

According to prescriptive decision theories, the generation of options for choice is a central aspect of decision making. A too narrow representation of the problem may indeed limit the opportunity to evaluate promising options. However, despite the theoretical and applied significance of this topic, the cognitive processes underlying option generation are still unclear. In particular, while a cued recall account of option generation emphasizes the role of memory and executive control, other theoretical proposals stress the importance of ideation processes based on various search and thinking processes. Unfortunately, relevant behavioral evidence on the cognitive processes underlying option generation is scattered and inconclusive. In order to reach a better understanding, we carried out an individual-differences study employing a wide array of cognitive predictors, including measures of episodic memory, semantic memory, cognitive control, and ideation fluency. The criterion tasks consisted of three different poorly-structured decision-making scenarios, and the participants were asked to generate options to solve these problems. The main criterion variable of the study was the number of valid options generated, but also the diversity and the quality of generated options were examined. The results showed that option generation fluency and diversity in the context of ill-structured decision making are supported by ideation ability even after taking into account the effects of individual differences in several other aspects of cognitive functioning. Thus, ideation processes, possibly supported by search and thinking processes, seem to contribute to option generation beyond basic associative memory retrieval. The findings of the study also indicate that generating more options may have multifaceted consequences for choice, increasing the quality of the best option generated but decreasing the mean quality of the options in the generated set.

## Introduction

Imagine that a friend asks for your advice. He is a member of a non-profit organization helping children. Unfortunately, public funding to the organization was cut and the organization members now need to find ways to raise money for supporting their activities. What would you suggest your friend to do?

This scenario is an example of a real-world decision-making situation in which the basic elements of the decision problems (options, evaluation dimensions, probabilities, and outcomes) are not explicitly specified and more than one valid solution exists. Following early problem solving research (see e.g., Reitman, 1964; Goel, 2010) these kinds of problems are considered ill-structured or only partially structured. In order to make a choice, individuals need to transform an ill-structured problem into a representation enabling a choice (e.g., Gettys et al., 1987; Finucane and Lees, 2005; Galotti, 2007). The process of decision structuring includes different aspects, like the identification of the viable options (option generation) and evaluation dimensions (attribute identification), as well as the definition of the potential outcomes and their associated values and probabilities (e.g., Frisch and Clemen, 1994; Parker and Fischhoff, 2005; Galotti, 2007). In the present paper, we will focus on option generation, which is a core aspect of decision structuring (Keller and Ho, 1988; Kalis et al., 2013).

Several scholars in decision making highlighted the importance of option generation and prescriptively warned about the perils of relying on a too-narrow problem representation, which may lead to the neglect of promising options (see e.g., Frisch and Clemen, 1994; Keeney, 1999; Hammond et al., 2002). However, decision-making researchers have traditionally spent very limited effort in the investigation of how problems are structured and options generated, compared to the effort devoted to the investigation of how people decide in completely-specified decision problems (e.g., choices between gambles, intertemporal choices, multi-attribute problems with full information, experience-based choices with well-identified alternatives). As a consequence, several aspects of option generation are still poorly understood.

Within the stream of studies on option generation in decision making, summarized in the next sections, the individual differences study reported in this paper had two main aims:

1. Contribute to the identification of the cognitive processes underlying option generation, disentangling the role of ideation processes from the role of relatively more basic cognitive processes (like associative memory retrieval and cognitive control);
2. Contribute to the clarification of the relationships between option generation fluency, quality of option generation, and choice quality.

In the next section, we will review the research on option generation relevant to each of these two goals, in order to provide the empirical and theoretical background of our work and the rationale for the hypotheses tested in our individual-differences study. Then, we will describe our study and its results. Finally, we will discuss the theoretical and applied implications of the findings, the limitations of the research, and delineate promising avenues for future investigation.

### Cognitive processes underlying the option generation process

The first goal of our study was to contribute to the identification of the cognitive processes underlying option generation through an individual-differences analysis. The limited number of studies carried out on option generation observed huge individual differences in the number of generated options (e.g., Manning et al., 1980; Engelmann and Gettys, 1985; Gettys et al., 1987). Some of these studies tried to identify the predictors of these individual differences in fluency in order to shed light on the processes underlying generation. They considered mainly expertise in the specific domains of investigation (e.g., car troubleshooting, geographical knowledge), general intelligence, memory, executive control, and ideation fluency or other measures of creative cognition. The findings suggest that expertise does not play a significant role, at least in the majority of investigations (e.g., Mehle, 1982; Klein et al., 1995, but see Engelmann and Gettys, 1985; Ward et al., 2011), intelligence, retrieval from long-term memory, and executive control seem to play some role (Manning et al., 1980; Channon and Crawford, 1999; Kaiser et al., 2013), while ideation fluency (i.e., the ability to produce various ideas in response to some preset requirements), as measured by the alternative uses test, seems to be an important predictor of performance, at least in rather complex problems (Manning et al., 1980; Engelmann and Gettys, 1985)^1^.

However, there are reasons to believe that these conclusions on the processes underlying option generation are not particularly convincing, thus motivating further studies on this issue. First, previous studies have adopted rather simple correlational and single-stage regression methods that did not allow a particularly strict control over the potential relations between predictors. Second, the rather small number and type of predictors considered in previous investigations may have neglected some important factors. Finally, the findings are not fully consistent across studies, with some investigations stressing retrieval from long-term memory and others highlighting the role of ideation fluency (e.g., Kaiser et al., 2013 vs. Engelmann and Gettys, 1985).

In relation to the last consideration, various theoretical explanations of option generation have been proposed, with a varying degree of specification. A first class of explanations conceives generation essentially as a cued recall process, supported by executive control or working memory (memory-based explanations). According to this view, option generation is based on the associative retrieval of options from long-term memory, triggered by elements of the problem (Adelman et al., 1995) and probably bounded by constraints related to the quality of the solution or to evaluative dimensions and usage contexts (Ratneshwar and Shocker, 1991; Johnson and Raab, 2003; Kaiser et al., 2013). Examples are the HyGene (Thomas et al., 2008) and the constrained retrieval models (Gettys and Fisher, 1979), with the former relying on a formal memory model (Dougherty et al., 1999) and the latter placing more emphasis on executive control processes orienting memory search. These two models focused more on hypothesis generation than on option generation, and they have been applied in rather constrained environments, where the space of potential options is usually more defined. Another memory-based account is the *Search for Ideas in Associative Memory* model (SIAM), proposed to explain individual idea generation in group settings (Nijstad et al., 2002; Nijstad and Stroebe, 2006). This model is based on memory associative retrieval within a localized set of information (image), leading to trains of semantically associated ideas. When retrieval fails, cognitively controlled operations are needed to find cues to access a different set of information (a different image) leading to different trains of thoughts^2^. The first hypothesis tested in our study stems from memory-based explanations of option generation: Individual differences in option generation fluency should be explained mainly by cognitive processes involved in associative cued recall and executive control, with ideation processes playing a marginal role or almost completely depending on more basic processes and thus not contributing further in terms of predictive capacity (*Hypothesis H1a: Memory as Predictor*).

A different class of explanations (henceforth, ideation-based models) conceives generation as a process not only rooted in basic associative retrieval and executive control but also requiring a significant ideation component (Keller and Ho, 1988; Goel, 2010), possibly supported by more complex search and thought processes. The findings of some studies suggest that these additional processes are needed to reach a good account of option generation fluency in less structured and more complex scenarios (e.g., Manning et al., 1980; Engelmann and Gettys, 1985). The ideation-based explanations differ in the mechanisms and strategies supposed to underlie idea generation, which range from the concept of leverage points as seeds for generation (rough ideas or opportunities leading to insightful outcomes and solutions)(Klein and Wolf, 1998), to the construct of lateral transformations (qualitative changes in ideation) as opposed to vertical transformations (more precise specification of a given idea) (Goel, 2010), and to a variety of strategies that can be used to generate ideas. These strategies include strategic modulation of activation in associative memory by focusing on different structural aspect of the problem (e.g., Keller and Ho, 1988; Keeney, 1994, 1999), ways for avoiding blocking ideas (e.g., Smith, 2003; Storm and Patel, 2014), analogical reasoning and context or perspective change (e.g., Keller and Ho, 1988; Ward et al., 1999). The analysis of these views leads to an alternative hypothesis tested in our study: at least in more complex and less constrained problems, individual differences in idea generation should be a significant predictor of individual differences in option generation fluency, over and beyond the influence of individual differences in more basic measures of cognitive ability (*Hypothesis H1b: Ideation as Predictor*).

Moreover, if option generation, unlike other decision-making tasks, depends on ideation ability rather than on other cognitive abilities (like cognitive control, memory, and fluid intelligence), then we should observe a behavioral dissociation between option generation performance and performance in other decision-making tasks relying on the other cognitive abilities but not requiring an ideative contribution (see also Kaiser et al., 2013). Thus, in order to allow a further test of the ideation hypothesis, we included the *Applying Decision Rules* subtest of the A-DMC battery (henceforth, ADR; Bruine de Bruin et al., 2007) in our study. Performance in this cognitively intensive decision-making task is related to individual differences in fluid intelligence (Bruine de Bruin et al., 2012), working memory (Del Missier et al., 2013), and executive control (Del Missier et al., 2012), but ADR does not require any kind of ideation. Thus, if the ideation hypothesis holds true, option generation fluency and ADR performance should show a behavioral double dissociation, meaning that ideation fluency will be positively related to option generation fluency but not to ADR performance, while the other cognitive measures will be positively related to ADR performance but not to option generation fluency (*Hypothesis H2: Behavioral Double Dissociation*).

### From option generation fluency to generation and choice quality

The second goal of our study was to contribute to the clarification of the relationships among option generation fluency, quality of option generation, and choice quality. The analysis of existing studies on option generation quality highlights an apparent disagreement apparent between scholars postulating that “less is more” (meaning that generating fewer options leads to a higher average quality of the option set and to better choices, Johnson and Raab, 2003) and others stating that “quantity breeds quality” (meaning that the more options generated, the better the quality of generated ideas will be—for reviews see e.g., Rietzschel et al., 2007; Nijstad, 2009) or at least pointing out clear limitations in typical option generation performance (Mehle, 1982; Gettys et al., 1987; Klein et al., 1995).

A number of studies carried out in diverse scenarios show that, despite the relatively small number of options generated by the “average participant,” at least some good options are usually reported (e.g., Gettys et al., 1987; Klein et al., 1995). Johnson and Raab (2003), analyzing action generation in handball players in a highly time-constrained task, highlighted the less-is-more effect in option generation, observing that the average quality of the option set declines with the increase in the number of generated options. They also showed that option quality tends to decline with the generation order (see also Klein et al., 1995) and found that generating and considering more options led to a less consistent and poorer choice (but see Ward et al., 2011, for contrasting findings). These results suggest that less can be more for the average quality of the option set, and that fast generation of some good options can be an effective strategy in environments in which it is critical to trade-off time and completeness of generation. This may hold also in other benign environments (Gettys et al., 1987), where a reasonably good or satisfying choice suffices and thus extensive search is not needed. Following this line of reasoning and related findings, a negative relation between option generation fluency and the mean quality of generated options was expected in our study (*Hypothesis H3a: Less is More*), together with a positive relationship between the mean quality of generated options and the quality of choice (*Hypothesis H4a: Mean Choice Quality*), because a higher mean quality of generated options structurally allows for a better choice.

On the other hand, several studies showed marked departures from the optimal generation performance, when optimal performance is measured by referring to the options with the highest utilities in different areas of the option space (e.g., Gettys et al., 1987). This happens in various domains, like solving a general parking problem in a campus (Gettys et al., 1987), finding an accommodation for an impecunious friend (Gettys et al., 1987), or identifying potential uses for a geographic area (Manning et al., 1980). Suboptimal generation has also been observed also in expert mechanics diagnosing automobile malfunctioning (Mehle, 1982) and in experienced chess players (Klein et al., 1995). These findings have been replicated even when providing very ample generation time or incentives for the quality or quantity of generated options (Gettys et al., 1987), suggesting a cognitively-driven difficulty in figuring out more options^3^. Thus, a too narrow and fast generation may not be appropriate in decision contexts in which making a very good choice is important, time constraints are not so strict, and the best options may not be so easily accessible due to lower familiarity or problem complexity. Finally, when the goal of the generation process is to find an original option (e.g., in marketing/advertising, scientific research, product design, art and literature), it is likely that limiting generation to the first accessible ones will lead to poor outcomes. In these cases, as studies in the social psychology and creativity arenas have shown (e.g., Parnes and Meadow, 1959; Diehl and Stroebe, 1987; Rietzschel et al., 2007), quantity can breed quality and a deeper and broader exploration of the option space can lead to better results. Thus, following the quantity-breeds-quality prediction, the option quality should increase with the number of generated options (*Hypothesis H3b: Quantity Breeds Quality*).

However, it can be also hypothesized that the less-is-more and quantity-breeds-quality co-exist and apply to different aspects of option generation performance, considering that less-is-more studies focused mainly on the mean quality variable, while quantity-breeds-quality investigations focused mainly on the max quality variable. Therefore, less-is-more may hold for the mean quality measure while quantity-breeds-quality effect may apply to the quality of the best option generated (*Hypothesis H3c: Co-existence of Effects*). Finally, as predicted for mean quality, a positive relation between max quality of generated options and choice quality can be expected (*Hypothesis H4b: Max Choice Quality*), because a higher max quality structurally allows for a better choice.

## Methods

### Participants

Participants were 141 undergraduate students (age: *M* = 22.74, *SD* = 3.24, 26% males). They participated on a voluntary basis. The APA ethical guidelines were followed in the realization of the study, with the study protocol approved by the Ethical Committee of the University of Trieste.

### Procedure

The study was carried out in two sessions. In the first one, lasting approximately 1 h, participants were tested in small groups. They received a booklet containing the tasks they had to complete, working individually under the experimenters' supervision. They first completed three different ill-structured option generation problems, generating as many possible options for solving each one as they could. In particular, the three option generation tasks required identifying possible options for solving a parking problem in the center of a middle-size city (after Gettys et al., 1987), a fund raising problem in a non-profit organization helping children, and a domestic energy saving problem at home (see the next section for a more detailed description). Written descriptions of these three problems were presented in sequence in fixed order and participants had 6 min for each problem to write down as many options they could figure out^4^. The instructions made clear that a short but precise description of each option was required. Each option had to be written on a new line of the response sheets following the scenario description. Participants were encouraged to keep searching for potential options even if they had the impression that they could not find other solutions. Experimenters timed the tasks. Soon after having completed each option generation task, participants chose the option they considered to be the best one for solving each problem among the ones they generated. After each option generation task, participants were asked some general questions about the task just completed. In particular, following previous studies, they rated perceived task difficulty, knowledge of and experience in the domain, and completeness of their performance on 7-point rating scales.

Following a short pause, participants completed the alternative uses test (Guilford et al., 1978; Gilhooly et al., 2007), generating as many alternative uses they could for a brick, a stable, and a tire, respectively. They were given 3 min for each object. After this task, they completed the six-item version of the Cognitive Reflection Test (Finucane and Gullion, 2010), and responded to a series of socio-demographic questions.

The second session of the study took place individually within 3 weeks from the first in a psychology laboratory of the university. During that session, participants completed several tests measuring individual differences in potential predictors of option generation performance (see next section for a more detailed description). In particular, they completed three tests of executive functioning (Letter-Memory, Stroop, Plus-Minus), a cued recall test of episodic memory, and tests of generation fluency in semantic memory (category and letter fluency). They also completed a fluid intelligence test (Raven's Standard Progressive Matrices: SPM) and the ADR subtest of the A-DMC battery. The order of the tests was as follows: Cued recall test (first session), Letter-Memory, Stroop, Plus-Minus, Cued recall test (delayed session), Raven's SPM, ADR, Letter Fluency, Category Fluency. A short break was allowed between each task, and a longer pause was placed approximately in the middle of the testing session, before Raven's SPM.

### Materials

#### Option generation tasks and scoring

Participants were first presented with each problem scenario in a written form and they were asked to generate as many valid options as they could. As anticipated, the three tasks involved generating potential options for solving a parking problem in the center of a middle-size city (after Gettys et al., 1987), a fund raising problem in a non-profit organization helping children, and a domestic energy saving problem (see Supplementary Material for the full text). These tasks are representative examples of ill-structured decision problems in the context of a study on option generation (where the general goal of the decision problem is usually given and the viable options are not). Moreover, these three problems were similar to the tasks employed by previous studies in the field (e.g., Manning et al., 1980; Engelmann and Gettys, 1985; Gettys et al., 1987). Parking problems affect many European cities and it seems reasonable to assume that they are relatively well-known to the population (e.g., Khattak and Polak, 1993), as well as the more common solutions that are available to policy makers and city authorities. Moreover, the parking domain has been used in previous option generation studies (e.g., Gettys et al., 1987; Adelman et al., 1995), and this allowed capitalizing to some extent on previous findings and methods. Fund raising in non-profit organizations is a less familiar domain for most individuals, and we selected it because we wanted to investigate also a less common type of problem^5^. However, all segments of the adult populations are targets of fund raising initiatives and all segments of the population (including undergraduates) are active in different kinds of non-profit organizations (Wilson, 2000). Thus, it is reasonable that a basic knowledge of this domain was available to our sample. Finally, energy saving problems represent a central issue in modern societies and they can be certainly considered as something relevant for most adult citizens. In this case, the individual can not only suggest viable options but also take personal decisions, which can have important consequences for the individual and the society (Gardner and Stern, 2002; Abrahamse et al., 2005). Education and massive media campaigns in the European Union have provided ample information on energy saving options in recent years. Thus, even in this case, it is reasonable to assume that a basic knowledge was available to the average undergraduate, even if not always accurate (Steg, 2008).

Following previous studies on idea generation (e.g., Diehl and Stroebe, 1987; Rietzschel et al., 2007) and option generation (Gettys et al., 1987), responses to each option generation problem were examined and scored by two judges, and this produced measures of generation fluency (number of options generated), diversity (number of different response category accessed), and quality (mean quality of the generated options, higher—max-quality of the generated options). For what concerns diversity, a measure capturing the ability to generate options from different areas of the option space, potential broad response categories (akin to the “limb” of the option threes in Gettys et al., 1987) were initially defined starting from previous studies and specific literature on the domain. In particular, for the parking problem, response categories were specified by considering the results of previous studies on option generation (Gettys et al., 1987) in the light of our specific problem, the parking plans of some medium-size Italian cities, and scientific and dissemination papers in the transportation and parking field (e.g., Marsden, 2006). For the fund raising problem, classifications were initially based on the financial and social budgets of several non-profit children organizations and humanitarian organizations (e.g., Save the Children). Finally, for the energy saving problem, the categories were created after the analysis of scientific and dissemination publications in the energy saving field (e.g., Gardner and Stern, 2002; Abrahamse et al., 2005; Coop, 2008). All the categories were then refined and adjusted after a pilot study. This led to the main response categories for the parking problem, the fund raising problem, and the energy saving problem^6^.

Two raters classified and rated the option generated by participants (e.g., Diehl and Stroebe, 1987; Rietzschel et al., 2007; Baas et al., 2011) after a short training. This included a first phase of selected readings on each domain (for examples, see the previous paragraph), and instructions explaining the classification procedure and taxonomies. Then, the raters underwent a classification supervised training with some examples of answers for each category. Then, each rater classified half of the options generated in each problem (belonging to a randomly selected half of the participants). Inappropriate responses (like “I don't care about this problem”) or too generic answers (i.e., “Tell the major to solve the parking problem”) were discarded (less than 2% of the total responses), and semantically negligible variations of the same responses (like “build new parkings outside the center” or “build new parkings in non-central areas”) were considered as the same item. Raters were then asked to evaluate each generated option on a 7-point scale (from *very low* to *very high*) for its potential utility in solving effectively the problem (quality). The inter-rater agreement of classifications was very good (*K* > 0.85 on a random sample of 20 participants), and the correlation between ratings for quality of responses was good (*r* > 0.70 in each problem). Pairwise average correlations between performance measures across the three generation scenarios were significant and positive for fluency (0.46, with the bivariate correlations *r*_park_fund_ = 0.35, *r*_park_ener_ = 0.50, and *r*_fund_ener_ = 0.52, all *p*s < 0.001) and diversity (0.19, with bivariate correlations *r*_park_fund_ = 0.24, *p* < 0.01, *r*_park_ener_ = 0.19, *p* < 0.05, but *r*_fund_ener_ = 0.15, *p* < 0.10), thus showing stability of individual differences in option generation across very different scenarios. The quality measures showed less stability across problems, with the correlation of the quality of the best option being significant between the parking and the energy saving problems only (*r*_park_ener_ = 0.17, *p* < 0.05).

#### Individual-differences tests

***Letter-memory***. The letter-memory task measures the ability to update working memory contents (e.g., Morris and Jones, 1990; Miyake et al., 2000). This ability was deemed relevant for our option generation task, due to the participants' need of updating working memory with new cues and retrieved information when continuing the search for new options. On the contrary, maintenance was not deemed so central because, in our option generation task, participants had to write down generated options, not to keep them in mind. However, it is worth noting that updating measures are generally rather strongly associated with capacity measures (see e.g., Schmiedek et al., 2009). In the letter-memory task, letters are presented in the center of the computer screen, one after another, with the length of the series varying randomly. At the end of the sequence, a message prompts participants to report the last three stimulus letters they have seen in the correct order. Thus, participants have to update working memory content in order to maintain an updated subset of stimuli. In our version of the task, following previous investigations (Del Missier et al., 2010, 2012), 14 series of letters were used, with the series length varying from 5 to 12 letters, and presented at the rate of 2 s per item. Two training series were also employed, to ensure that participants correctly understood the task instructions. The task was presented electronically using the E-Prime 2.0 software (Psychology Software Tools, Pittsburgh, PA). We used as the final score the proportion of triples correctly reported. Reliability, computed with Cronbach's alpha, was 0.44.

***Stroop***. We used a manual-response version of the Stroop task (Stroop, 1935) to measure the ability to inhibit a prepotent response (see e.g., Miyake et al., 2000, see Del Missier et al., 2010, 2012). This task was included in our study because the inhibition of old options or stereotyped responses may be relevant in option generation. In this task, a set of three words is presented on the screen in 96 trials, with the central word being colored (in red/blue/green/yellow) and the lateral ones always printed in black. In half of the trials the color and the central word are congruent (e.g., the word “red” is printed in red), whereas in the other half, they are incongruent (e.g., the word “red” is printed in blue). The two lateral words, printed in black, are two different color names (red/blue/green/yellow), but only one matches the color of the central world and the other one is a foil. The participant has to press a right-side key to indicate that the color of the central word corresponds to the color name on the right side of the screen and a left-side key to indicate that the color corresponds to the color name on the left side. The task was administered via an E-Prime 2.0 script, with a feedback-enabled training phase of six trials. The performance score for this task was the Stroop RT (difference in mean RT between incongruent trials and congruent trials). Reliability, computed with the split-half (odd-even) correlation adjusted by the Spearman-Brown prophecy formula, was 0.76.

***Plus-minus***. This task measures the ability to shift between tasks (Jersild, 1927; Spector and Biederman, 1976; Miyake et al., 2000), which may be relevant in option generation, considering the need to switch between response categories. In the Plus-Minus task, the participants have to add three to each of a first series of 30 numbers, and then they subtract three from each of another series of 30 numbers. Finally, they have to shift between summing and subtracting three from each of a third series of 30 numbers. The task score is computed as a shift-cost measure, by taking the difference between the reaction time (RT) needed to complete the final alternating series and the mean RT across the first two series. The plus-minus task was administered twice to our participants, using two different series of numbers (Del Missier et al., 2010, 2012). Before the first administration of each component of the task, participants underwent a short practice with a reduced series of numbers. The final score was the average of the shift-cost measure across the two trials. Reliability, assessed by the correlation between the two alternate trials, was 0.82 (*p* < 0.001), while switch cost reliability was 0.41 (*p* < 0.001).

***Paired-associates cued recall test***. This task measures the ability to retrieve items from long-term episodic memory after the provision of associated cues. We employed a cued recall test because memory-based explanations generally assume that a cued recall process underlies option generation (see Introduction). In the encoding session of this test, 20 word pairs were presented, including strong and weak associates (e.g., key-door, spade-guilty). Stimuli were drawn from the Italian version of the Wechsler memory scale (Wechsler, 1997). Participants were asked to learn the word pairs in view of a cued retrieval test (i.e., retrieve the second word after being presented with the first). An E-Prime 2.0 script presented each word pair for 5 s in the center of the computer screen, with the order of pairs randomized. Thirty seconds after the end of the learning session, filled with an interpolated task, participants completed a first cued recall test. In this test, the first word of each pair was presented as a cue (in a different random order, fixed for all participants), asking the participant to report its paired associate within 5 s. After 30 min, filled with other tests in the battery, participants were tested again, using a different random order of cues for the delayed test. Performance in both paired associates tests (immediate and delayed) was assessed by the number of correctly retrieved words. Reliability, assessed by the correlation between the immediate and the delayed versions, was 0.93 (*p* < 0.001).

***Category and letter fluency tests***. Verbal fluency tests are semantic memory test that may also require working memory and executive control (Rende et al., 2000; Gilhooly et al., 2007; Del Missier et al., 2013). Their inclusion in our study was motivated by the fact that these tasks require generation from memory, and thus verbal fluency tests tap memory processes that may be very similar to the ones involved in option generation. Following previous research, our instructions included the indication to avoid producing person names (e.g., “S” → Sarah) or minor variations of the same words (e.g., transportation means → “cars” after having previously generated “car”). Before the testing phase, we also ran two short training sessions, which consisted in generating, within 1 min of time, all the possible words indicating transportation means (category fluency) or beginning with the letter B (letter fluency). After the practice phase, participants underwent two category fluency sessions (Animals, Fruits), and two letter fluency sessions (S, F). Each test session had a time limit of 2 min. Participants' oral responses were recorded and subsequently transcribed. Then, all valid responses were counted, and the word count was used as the performance score. Reliabilities, as measured by the correlations between the two category trials and two letter trials, were 0.43 (*p* < 0.001) and 0.71 (*p* < 0.001), respectively.

***Raven's standard progressive matrices (SPM)***. In Raven's SPM, a widely used test of fluid intelligence (Raven et al., 2003), participants are asked to select, from an array of figures, the one that completes a given pattern or sequence. This test is related to cognitively demanding measures of decision-making competence (e.g., Bruine de Bruin et al., 2007; Del Missier et al., 2012) and this motivated its use in our study. Raven's SPM presents 60 patterns of increasing difficulty in five series. In the present study, following previous research (see e.g., Friedman et al., 2006; Bruine de Bruin et al., 2007), we used half of the stimuli, randomly selected within each series (all odd or all even items). This usually allows achieving a good assessment of fluid intelligence while keeping shorter the administration time. The performance score was the number of correct answers. Reliability, assessed with Cronbach's alpha, was 0.68.

***Applying decision rules (ADR)***. This A-DMC task (Bruine de Bruin et al., 2007) assesses the ability to apply accurately decision rules. It was included as a criterion task to test our behavioral double dissociation hypothesis (H2), considering that ADR performance is positively related with measures of working memory, fluid intelligence, and executive control but the task does not require any form of ideation (see Introduction). Each item of the task (see Supplementary Material for one example) requires the participant to select one or more options from a table displaying five options described on five attributes. The task presents participants with 10 different multiattribute choices between DVD players with varying numerical ratings on different features (such as sound quality). For each problem, the participants are asked to follow a specific decision rule (e.g., lexicographic, satisficing), verbally described to them, in order to select one or more options. Final scores reflect the percentage of correct responses across items. Cronbach's alpha for the task was 0.56.

***Cognitive reflection test (CRT)***. This task was designed to measure analytical thought ability (Frederick, 2005), which is relevant in several high-level cognitive tasks. Each item is a mathematical word problem that triggers an intuitive but wrong answer. The participants must block that answer and substitute it with a correct one (Kahneman and Frederick, 2007). The task requires executive control and numerical competence (Del Missier et al., 2012). We used the six-item version of the CRT (Finucane and Gullion, 2010). The overall score was the number of correct answers across items. Reliability, assessed with Cronbach's alpha, was 0.73.

***Alternative uses test***. The alternative uses test is the more commonly used measure of ideation ability (Guilford et al., 1978; Gilhooly et al., 2007), and it proved to be a good predictor of option generation performance (Manning et al., 1980; Engelmann and Gettys, 1985). In our application, following previous studies, participants had to specify as many alternative uses they could for a brick, a staple, and a tire, with 3 min of time allotted for each object. The task was timed and written answers had to be provided. Fluency scores for each trial were obtained by counting the number of alternative responses produced by participants, which is the main measure used to score this task. Considering that originality was not a dimension of interest in our option generation study (see also Footnote 1), we focused only on fluency scores. The average correlation between the ideation fluency measures across the three problem scenarios was 0.53, with the bivariate correlations all positive and significant (*p* < 0.001).

### Overview of statistical analysis

We carried out two sets of analyses, related to the two main aims of the study. The first set of analyses tested hypotheses on the cognitive processes underlying option generation via correlations, hierarchical regression, and multiple regression. In particular, a hierarchical regression was employed to test the memory vs. ideation hypotheses (H1a vs. H1b) on their capacity to predict option generation fluency and diversity in each of the three decision-making problems. As a further assessment of the ideation hypothesis, we also tested, by using multiple regression, the predicted behavioral double dissociation between ADR performance and option generation performance (H2).

The second set of analyses tested hypotheses on the relationships among option generation fluency, quality of option generation, and choice quality. This was done via correlations and structural equation modeling. First, for each decision problem, we computed correlations and partial correlations between fluency/diversity and quality scores as a first comparative test of the less is more hypothesis (H3a), the quantity breeds quality hypothesis (H3b), and the co-existence of effects hypothesis (H3c). With the same methods, we also tested the two hypotheses on choice quality (H4a and H4b). Then, as a more sophisticated test of hypotheses H3 and H4, we specified and tested a path analysis model. The model incorporated both less-is-more and quantity-breeds-quality predictions, as directed relations from fluency to mean and max quality of generated options, respectively (H3). It also included the two postulated relations between generation quality measures and choice quality (H4). Finally, using path analysis estimates, we assessed the indirect effects of option generation fluency on choice quality via option generation quality measures. Specific methodological details are provided before each analysis.

## Results

### Predictors of option generation fluency

For what concerns option generation fluency and diversity, a descriptive summary of the results is presented in Supplementary Material (Table 1A). In line with previous studies, the range of fluency and diversity values shows large individual differences, thus justifying an individual-differences analysis. Individual performance ranges from the access to just one response category and the production of two valid options to the generation of 6/7 times more options in almost all (or all) the response categories. Moreover, in order to account for the observed variation in performance across problems, we carried out separate analyses for each decision problem.

The analysis of Pearson's bivariate correlations (Table 2A in Supplementary Material) shows moderate positive correlations between measures of ideation fluency (i.e., alternative uses test scores) and measures of option generation fluency and diversity in all the decision problems. Measures of episodic memory, cognitive control, verbal fluency, fluid intelligence, and analytical thought are generally not correlated with option generation fluency and diversity measures, with the exception of the energy saving problem. Experience and domain knowledge seem to play some role only in relation to the more familiar energy saving domain. The correlations with ADR show the opposite pattern: performance in this task is not significantly related with measures of ideation fluency but related with measures of episodic memory, cognitive control, verbal fluency, fluid intelligence, and analytical thought. These findings provide a first support for the ideation hypothesis (H1b) and a partial support for the behavioral dissociation hypothesis (H2). However, further steps are needed, considering that the relationship between ideation fluency scores and option generation measures may depend on the cognitive process underlying both tasks (for instance, memory and control processes).

To show that ideation fluency supports option generation beyond more basic cognitive measures, we carried out a hierarchical regression analysis for each problem with option generation fluency and diversity as criterion variables. In this analysis, we included in the set of predictors four compound variables made from the cued recall measures, the category fluency scores, the letter fluency variables, and the ideation fluency scores, respectively. This was done by transforming the raw measures in standardized scores and by averaging these (unweighted) scores to form a compound variable for each construct (cued associative memory, category fluency, letter fluency, and ideation fluency). Then, to establish a particularly strict test of our hypotheses, we included in the regression model the verbal fluency compound variables *before* the ideation fluency variable. Considering that verbal fluency tests, the alternative uses test, and option generation all require generation, they may share variance related to common method or common underlying process. Including into the hierarchical regression verbal fluency before ideation fluency means that this common variance will be explained by verbal fluency, thus allowing a “purer” assessment of the predictive contribution of ideation fluency to option generation. To the best of our knowledge, no previous study on individual differences in option generation applied this strict form of control. Moreover, the order of other predictors in the hierarchical regression analysis was chosen in order to carry out the stricter possible test of the ideation hypothesis, while including, at the same time, more basic cognitive measures (like executive control and cued recall) before measures that can depend on them (verbal fluency, and knowledge/experience).

In the first step of hierarchical regression we entered all the basic cognitive measures (executive functioning tests, cued recall compound, Raven's SPM, CRT), with the exclusion of the generation-based ones. In the second step we included verbal fluency compound variables (category and letter fluency). In the third step, we entered measures of domain knowledge and experience. In the final and fourth step, we added the ideation fluency compound variable. The results of hierarchical regression for option generation fluency are showed in Table 1. Results for diversity are very similar and thus they are not reported^7^. However, they are available on request.

**Table 1:** **Hierarchical regression results**.

**Problem**	**Step**	**Predictors**	***R*^2^**	**Δ*R*^2^**	**Δ*F*, *df*, *p* Δ*F***
Parking	1	Basic cognitive measures^a^	0.019	0.019	Δ*F* = 0.411, *df* = 6, 128, *p* = 0.871
	2	Verbal fluency measures^b^	0.037	0.018	Δ*F* = 1.173, *df* = 2, 126, *p* = 0.313
	3	Knowledge, experience	0.062	0.025	Δ*F* = 1.676, *df* = 2, 124, *p* = 0.191
	4	Ideation fluency^c^	0.255	0.193	Δ*F* = 31.778, *df* = 1, 123, *p* < 0.001
Fund Raising	1	Basic cognitive measures^a^	0.025	0.025	Δ*F* =0.536, *df* = 6, 128, *p* = 0.780
	2	Verbal fluency measures^b^	0.036	0.011	Δ*F* = 0.749, *df* = 2, 126, *p* = 0.475
	3	Knowledge, experience	0.048	0.012	Δ*F* = 0.764, *df* = 2, 124, *p* = 0.468
	4	Ideation fluency^c^	0.189	0.141	Δ*F* = 21.443, *df* = 1, 123, *p* < 0.001
Energy Saving	1	Basic cognitive measures^a^	0.052	0.052	Δ*F* = 1.167, *df* = 6, 128, *p* = 0.328
	2	Verbal fluency measures^b^	0.094	0.042	Δ*F* =2.900, *df* = 2, 126, *p* = 0.059
	3	Knowledge, experience	0.156	0.062	Δ*F* = 4.550, *df* = 2, 124, *p* = 0.012
	4	Ideation fluency^c^	0.323	0.168	Δ*F* = 30.525, *df* = 1, 123, *p* < 0.001

a *Basic cognitive measures: executive functioning tests, cued recall compound variable, Raven's SPM, CRT*.

b *Verbal fluency measures: category fluency and letter fluency compound variables*.

c *Ideation fluency: ideation fluency compound variable*.

The results of hierarchical regression show that ideation fluency is the strongest predictor of option generation fluency (and diversity) and highlight its predictive contribution over and beyond the contribution of other cognitive factors. This holds for each of the three problems investigated. However, in the energy saving problem, a smaller contribution from knowledge/experience and verbal fluency is apparent. Overall, the regression models explain approximately between 20 and 30% of the variance in each problem. These findings provide clear support for the ideation hypothesis (H1b). Disaggregating the cued recall compound variable from the other basic cognitive measures and entering it in a separate regression step did not change the pattern of findings. The only minor change was observed in the energy saving problem, with cued recall (Δ*R*^2^ = 0.024, *p* = 0.075), verbal fluency (Δ*R*^2^ = 0.042, *p* = 0.059), and knowledge/experience (Δ*R*^2^ = 0.062, *p* = 0.012) all showing marginally significant or significant effects, but ideation fluency still contributing more than the sum of these effects (Δ*R*^2^ = 0.168, *p* < 0.001). The same happened when cued recall was entered alone in the first regression step (Δ*R*^2^ = 0.034, *p* = 0.032), with ideation fluency still contributing more than the sum of all the other significant effects (Δ*R*^2^ = 0.168, *p* < 0.001).

As a test of the behavioral double dissociation hypothesis (H2), we specified alternative regression models in which option generation performance and ADR scores were selectively predicted by the ideation fluency compound variable vs. all the other cognitive measures. The results of these analyses are showed in Table 2. The multiple regression results for diversity were very similar and thus they are not reported here. The same holds for a two-step hierarchical regression carried out in order to control for common method variance (i.e., the ideation fluency compound variable was entered after the verbal fluency compound variables). However, these results are available on request.

**Table 2:** **Multiple regression results**.

**Criterion**	**Model**	***R*^2^**	**ANOVA**	**Significant predictors (standardized coefficients)**
Parking	Basic cognitive model^a^	0.037	*F*_(8, 127)_ = 0.607, *p* = 0.770	None
Option generation fluency	Ideation fluency model^b^	0.212	*F*_(1, 138)_ = 37.139, *p* < 0.001	Ideation fluency 0.460^***^
Fund raising	Basic cognitive model^a^	0.036	*F*_(8, 127)_ = 0.592, *p* = 0.783	None
Option generation fluency	Ideation fluency model^b^	0.144	*F*_(1, 138)_ = 23.255, *p* < 0.001	Ideation fluency 0.380^***^
Energy saving	Basic cognitive model^a^	0.094	*F*_(8, 127)_ = 1.639, *p* = 0.120	Category fluency 0.201^*^
Option generation fluency	Ideation fluency model^b^	0.234	*F*_(1, 138)_ = 43.448, *p* < 0.001	Ideation fluency 0.489^***^
Applying decision rules	Basic cognitive model^a^	0.365	*F*_(8, 127)_ = 9.144, *p* < 0.001	Letter memory 0.160* Raven 0.293^***^ CRT 0.223^**^ Letter fluency 0.142^∧^
	Ideation fluency model^b^	0.006	*F*_(1, 138)_ = 0.755, *p* = 0.380	Ns

Two-tailed significance levels:

*** p < 0.001;

** p < 0.01;

* p < 0.05;

∧ *p < 0.10*.

a *Basic cognitive model: executive functioning tests, cued recall compound variable, Raven's SPM, verbal fluency compound variables, CRT*.

b *Ideation fluency model: ideation fluency compound variable*.

The findings in Table 2 show a double dissociation between option generation predictors and ADR predictors in two of the problems considered (parking and fund raising). In the third problem, category fluency seems to play a role in option generation (see also the hierarchical regression results in Table 3), but the stronger predictor remains ideation fluency. Overall, these results suggest that ADR and the option generation tasks used in the present study rely on rather different cognitive processes, with the former being more based on control, memory, and analytical reasoning processes (see also Bruine de Bruin et al., 2007; Del Missier et al., 2010, 2012, 2013) and the latter being more dependent on ideation skills. Thus, they seem to represent different facets of decision-making competence.

**Table 3:** **Pairwise bivariate correlations between measures of option generation**.

	**Parking**		**Fund raising**		**Energy saving**	
	**Mean quality after controlling for max quality**	**Max quality**	**Mean quality after controlling for max quality**	**Max quality**	**Mean quality after controlling for max quality**	**Max quality**
Fluency	−0.18^*^ (−0.13)	0.05	−0.21^*^ (−0.02)	0.27^**^	0.03 (0.16 ^∧^)	0.31^***^
Diversity	−0.05 (−0.02)	0.04	−30^***^ (−0.12)	0.22^**^	0.26^**^ (0.37^***^)	0.32^***^
Choice quality	0.30^***^ (0.42^***^)	0.36^***^	0.28^**^ (0.34^***^)	0.21^*^	0.45^***^ (0.50^***^)	0.23^*^

Two-tailed significance levels:

*** p < 0.001;

** p < 0.01;

* p < 0.05;

∧ *p < 0.10. Correlations involving mean quality are partial correlations, after controlling for the respective max quality ratings. Non-partial pairwise correlations are reported in parentheses*.

### Relationships between fluency, option generation quality, and choice

Descriptive statistics and 95% confidence intervals for quality measures, presented in Supplementary Material (Table 3A), show that participants did a good option generation job in each decision problem. Clear individual differences in mean and max quality variables, as seen in range and variability data, justify an individual-differences analysis.

Table 3 shows Pearson's bivariate correlations between fluency/diversity and quality measures. Considering that the mean quality scores include also the rating of the best option generated, in the case of mean quality we report partial correlations after controlling for max quality, in order to disentangle the specific effects of fluency/diversity on mean quality and their consequences for choice.

The findings fully support the choice quality hypotheses (H4a and H4b) and they tell us that a better generation fosters better choices. A better choice quality seems to be related both to a higher mean quality of the generated option set (after controlling for max quality) and to the quality of the best option generated. There is also evidence compatible with a less is more effect (H3a) in the parking and fund raising problems and for the mean quality variable. On the other side, the analysis of max quality provides evidence compatible with a quantity breeds quality view (H3b) in the fund raising and energy saving problems (but not in the parking problem). To summarize, the overall picture of findings seems more compatible with a co-existence of effects hypothesis (H3c) than with the two other hypotheses (H3a and H3b), with negative correlations between fluency and quality apparent in the mean quality measures and positive correlations apparent in the max quality scores, even if some findings in the parking and energy saving problems are not consistent with H3c. Thus, an appropriate summary of the findings is that higher fluency and diversity had both positive and negative effects on generated option quality, with the positive effects seen mainly in the quality of the best generated option and the negative ones seen in the average quality of the other options generated in the set, but with these effects also being moderated by the problem.

**Figure 1:**
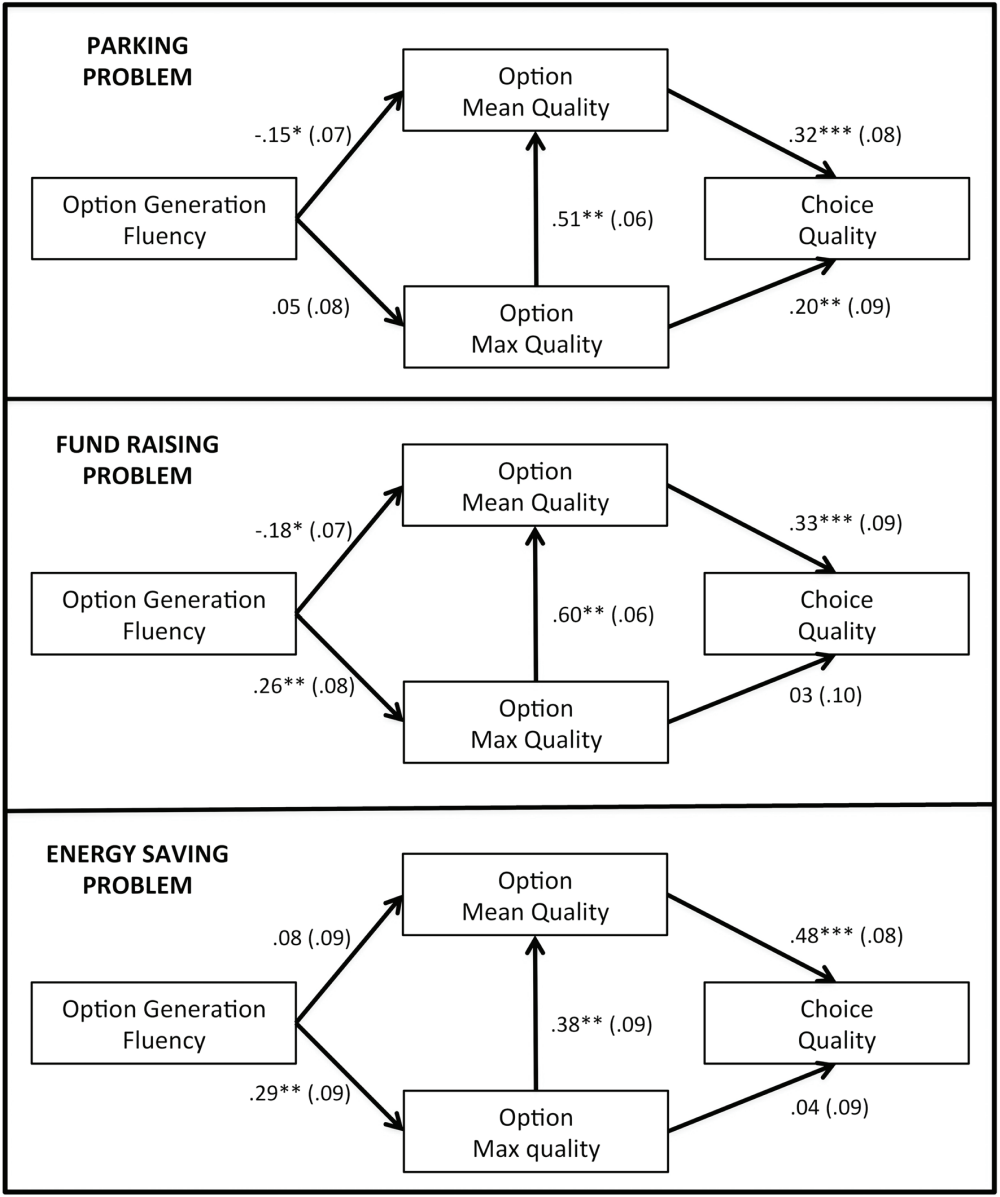
**Path analysis model for each problem**. Note: Two-tailed significance levels: ^***^p < 0.001; ^**^p < 0.01; ^*^p < 0.05. The standard error for each standardized path coefficient is reported in parentheses.

Correlations between option generation fluency and choice quality were all non-significant (parking: *r* = −0.01; fund raising: *r* = 0.08; energy saving: *r* = 0.14), but Table 3 suggests the existence of indirect effects of fluency via mean quality or max quality. Thus, in order to shed further light on the network of relationships linking option generation fluency, generation quality, and quality of choice, we estimated these relationships via path analysis. In particular, starting from H3 and H4, we specified a path-analysis model and used it for further testing in each problem. The model includes the relation between fluency and mean option quality (less is more), the relation between fluency and max quality (quantity breeds quality), the two relations between the option generation quality measures and choice quality, and the structural relation between mean and max quality (Figure 1). The model was estimated in each decision problem by using the Sepath module of the Statistica 12 software (version 12, StatSoft Inc., Tulsa, OK), starting from correlation matrices and using the maximum likelihood method. Model fit is reported in Table 4.

**Table 4:** **Fit for the path analysis model in the three decision problems**.

**Model fit indices**	**Parking problem**	**Fund raising problem**	**Energy saving problem**
Chi-square, *df, p*	0.123, 1, 0.726	1.332, 1, 0.248	0.277, 1, 0.599
RMSR	0.008	0.027	0.013
CFI	1.000	0.996	1.000
RMSEA	0.000	0.048	0.000
APGI	1.000	0.988	1.000

All the path analysis models showed very good fit indices. The analysis of standardized coefficients generally agrees with correlational analysis, showing both less is more effects of fluency on mean quality and quantity breeds quality effects of fluency on max quality. Moreover, choice quality is positively related with mean quality in all the problems, and with max quality in the parking problem. As the final step, we computed the indirect effects of fluency on choice (Table 5). We tested them via one-tailed Sobel tests for two-paths effects (Sobel, 1982) and the joint significance test for the three-paths effect (see e.g., Taylor et al., 2008).

**Table 5:** **Indirect effects of fluency on choice quality**.

**Indirect effect**	**Parking problem**	**Fund raising problem**	**Energy saving problem**
Fluency → mean quality → choice quality	−0.05^*^	−0.06^*^	0.04
Fluency → max quality → choice quality	0.01	0.01	0.03
Fluency → max quality → mean quality → choice quality	0.01	0.05^*^	0.05^*^

Significance level:

* *p < 0.05*.

The findings confirm that, in two of the problems scenarios, generating more options had a negative indirect effect on choice quality (less is more), possibly because generating more and more options lowers the average quality of the generated option set. However, at the same time, generating more options increased the value of the best option in two of the problems (quantity breeds quality). This means that it may promote the discovery of some high quality options even if other generated options are not so good. This may foster a better choice if the decision maker is able to select the best option. These opposite effects may also occur in the same problem. However, the indirect effects observed in our study are of small magnitude.

## Discussion

The individual-differences study on option generation reported in this paper aimed at contributing to the identification of the cognitive processes underlying option generation and to the clarification of the relationships between option generation fluency, quality of option generation, and choice quality. We will discuss our findings in relation to previous research on these two main issues. Then we will discuss the limitations of our research and outline future research directions.

### Predictors of option generation and underlying processes

We investigated the cognitive processes underlying option generation via an individual-differences study that considered a wider array of cognitive predictors and decision problems than previous studies. This allowed a more informative analysis that overcame some of the limits of earlier research. We can summarize the results of our investigation in two main points.

First, we found that individual differences in ideation ability contribute to the prediction of option generation fluency and diversity measures well-beyond the (limited) predictive capacity of more basic measures of executive control, episodic memory, semantic memory, fluid intelligence, and knowledge/experience of the domain. This held in all the decision scenarios we considered, even if some problem-related variation was apparent, with memory- and experience-related measure playing a significant role in the more familiar energy saving scenario. Although the potential role of ideation fluency has been recognized by some pioneering studies (Manning et al., 1980; Engelmann and Gettys, 1985), previous investigations had not been able to exclude the possibility that this influence could be entirely traced back to more basic processes of associative retrieval, executive control, or fluid intelligence. Moreover, rather surprisingly, the ideation component almost disappeared from the research agenda on option generation after these pioneering studies. Thus, our results represent a step forward in the decision-making literature on option generation.

Second, our findings showed that the ability to generate more options and more varied options in response to poorly-structured problems appears to be a clearly distinct aspect of decision-making competence from the ability to apply given choice rules to well-structured multi-attribute decision problems (see also Goel, 2010; Kaiser et al., 2013). Although option generation is usually considered as a distinct facet of decision-making competence by some theories (e.g., Finucane and Lees, 2005—and a similar argument has been posited in ill-structured problem solving, see Goel, 2010), no previous behavioral investigations have been able to provide clear empirical evidence supporting these theoretical stances. The provision of such evidence is our second step toward a better understanding of decision-making skills.

The first theoretical implication of our study is that models of option generation based only on cued recall seem unable to explain the pattern of results we obtained and, in particular, to account for the role of ideation fluency in option generation. Thus, these models, if applied to the same kinds of realistic and rather complex scenarios we investigated, need to be extended to include the influence of more complex thinking and search processes that people probably use to generate options. Even if past research provides some hints on these potential processes and strategies (e.g., Keller and Ho, 1988; Ward et al., 1999; Smith, 2003; Goel, 2010), their nature is far from being elucidated, and future work should shed more light on them, for instance by using process tracing methods like concurrent verbal protocols (Goel et al., 2013), smart experimental paradigms derived from the logic of the process dissociation approach (Jacoby, 1991), and scaling up existing neuroimaging paradigms to more complex and realistic decision-making scenarios. This will require new theoretical efforts and empirical studies, and it may open a new promising stream of research. In particular, future research should shed light on the possible interactions between retrieval and ideation processes, explaining how retrieval supports ideation and showing what kinds of ideation strategies are used in different problems and circumstances. Even if there are reasons and evidence to believe that memory and control process may support idea generation (e.g., Gilhooly et al., 2007), our study shows that ideation process in the context of option generation cannot be traced back entirely to these relatively more basic cognitive processes. A more detailed analysis of thinking and search processes is thus needed to understand what are the relations linking more basic retrieval and control processes, thinking and search strategies involved in ideation, and option generation outcomes.

Another interesting implication of our study is that the degree of reliance on associative retrieval or memory recall may depend on the problem or problem domain. A similar observation seems to hold for experience and knowledge of the domain. It is likely that associative processes capitalizing on previous knowledge and experience suffice to provide good options in familiar problems (e.g., deciding where to have lunch today in your campus), while a much greater role is played by the ideation thought process when finding out solutions in non-routinary contexts (e.g., identifying a valid solution for the non-profit fund raising problem), but perhaps also in more familiar ones when novelty is a requirement (e.g., devising an intriguing title for your next paper). Therefore, another goal of future research may consist in assessing systematically how variation in the type of problem, in relation with domain knowledge and experience, affects the degree to which option generation depends on control and retrieval processes vs. ideation processes (see also Kaiser et al., 2013).

A final theoretical aspect is that option generation in less familiar contexts seems to recruit different processes than choice by description, or choice by experience, where the options are well-specified. Our findings provide a well-fitting behavioral complement to the proposal of a functional and, possibly, neural dissociation between processes underlying preliminary option generation in ill-structured complex decision making and processes involved in more structured decision-making processes. In this regard, option generation can be considered as a different facet of decision-making competence, requiring ideation skills and thought abilities not required in well-structured decision tasks. This suggests the possibility that other aspects of decision structuring, for instance attribute identification or generation, may require similar skills and, more in general, it motivates efforts aiming at a better theoretical and empirical characterization of decision structuring skills. It also supports the idea that decision-making competence should be better viewed as a multifold construct, composed of partially distinct abilities that rely differentially on diverse cognitive and non-cognitive abilities (Finucane and Lees, 2005; Strough et al., 2011, 2015; Del Missier et al., 2013, 2015).

On the applied side, our findings suggest the need to design and validate measures and instruments that can be used to assess individual differences in option generation, building on our current work, previous work (e.g., Gettys et al., 1987), and neuropsychological research (e.g., Channon and Crawford, 1999, 2010; Channon, 2004; Goel et al., 2013). These instruments can be added to the existing repertoire of decision-making competence measures (Parker and Fischhoff, 2005; Bruine de Bruin et al., 2007; Finucane and Gullion, 2010) and they will allow the assessment of a broader range of decision-making skills. Existing batteries may be currently missing a central component of decision-making competence, which may be related to important aspects of real-world performance, especially in less familiar contexts or when solution diversity and novelty are important. Moreover, considering the individual's limitations in option generation in the more complex and less familiar situations^8^, especially if variety is also sought, researchers should devise smart ways to help the decision makers in these contexts. These helps may build, for instance, on the power of nominal group generation (e.g., Gettys et al., 1987; Nijstad, 2009) or on recommendation technologies that capitalize on huge bases of data about the users and the options in the domain (e.g., Burke et al., 2011).

### Relationships between fluency, option generation quality, and choice

The analysis of the relationships between option generation fluency and the quality of generated options highlighted both less is more effects and quantity breeds quality effects, generally related to different aspects of performance (mean quality and max quality of generated options, respectively). In this case, however, the results were not completely consistent across problems. We also observed that both the mean quality and the max quality of options in the generated set can affect choice quality, and that option generation fluency has indirect effects on choice quality.

For what concerns less-is-more effects, it is interesting to observe that we found negative relationships between option generation fluency and mean quality of generated options even in much less time-constrained problems than the ones in which these effects were originally found (e.g., Johnson and Raab, 2003). This shows that less-is-more effects can be observed even in the absence of strict time constraints, and our analyses confirmed that they may have consequences for choice. However, as already remembered, the effect was modulated by the problem. Thus, future studies should investigate its generality. A possibility, suggested by our findings, is that participants may be less affected by the effect in the more familiar and known domains, in that experienced individuals may generate a set of options that are all generally good and thus their mean quality may not be so strongly affected by moderate variations in fluency.

At the same time, we also observed quantity-breeds-quality effects in our study. These effects are generally not an object of investigation in the option generation research, although they are usually found in idea generation studies (Diehl and Stroebe, 1987; Rietzschel et al., 2007). Interestingly, the quantity-breeds-quality effects were observed mainly in relation to the max quality measure, which suggests the intriguing possibility that less-is-more and quantity-breeds-quality effects may coexist. This could contribute to reconcile diverging views in the literature on generation (e.g., Johnson and Raab, 2003 vs. Gettys et al., 1987; Diehl and Stroebe, 1987), suggesting that apparently opposite effects may apply to different facets of performance and, more generally, unveiling a further relation between studies on option generation and research on idea generation. However, as we have observed, these effects show variation across problems, and thus their influence on choice quality may depend on the problem at hand. This further motivates a more systematic investigation of the influence of problem features and decision-maker's knowledge.

### Limitations and future research directions

We would like to point out some limitations of the current study that may suggest new directions for future research. First, our sample was mainly composed of young and educated undergraduates, and this may have limited the range of individual differences we observed. Even if the results clearly show that individual differences are sizable in our sample, carrying out further studies on a more heterogeneous and varied sample of the population will probably show even stronger effects and it will allow an assessment of the external validity of our findings. As a related topic, the present investigation could be expanded to cover the adult life span, thus shedding more light on age-related changes in option generation (e.g., Del Missier and Terpini, 2009).

A second limitation is related to the set of predictors we employed. Even if we included a larger and more varied set of predictors than previous studies, the list is non-exhaustive and other abilities and personal characteristics potentially relevant for option generation may be included in future investigations (such as comprehension skills, different measures of ideation skills, need for cognition and closure, openness to experience). Moreover, future studies may consider additional memory measures, like the ability to inhibit or forget memories and thoughts (e.g., Storm and Patel, 2014), or free recall tasks that may be better able to capture individual differences in self-structured retrieval processes, and even multiple measures for each construct within a latent variable approach (Miyake et al., 2000; Del Missier et al., 2012, 2013).

Other limitations are related to the methods employed. Even if our findings are in line with previous research on similar problems (Manning et al., 1980; Gettys et al., 1987), and we followed established methods for this type of study, varying some aspects of data collection and scoring may be useful to evaluate the robustness of our findings. For instance, it may be worth extending the time available for generation (although time was already rather long when compared to some of the previous studies; see e.g., Johnson and Raab, 2003; Kaiser et al., 2013). Additionally, it can be useful to collect also expert ratings of the quality of the generated options. Although the reliability of our trained raters was good and Gettys et al. (1987) found nearly identical estimates of option utility when employing expert and non-expert ratings in a parking problem, using also experts ratings would strengthen our findings.

Another potential methodological issue may concern the findings on the mean quality of generated options when the influence of the max quality is statistically controlled for. In this case, after the effect of the best option has been partialed out, this effect relates to the mean quality of the other options in the set. Although, in our opinion, this control is needed for a test of the less-is-more vs. quantity breeds quality effects, it is worth pointing out that, without this control, no significant correlations between fluency/diversity and mean quality would have been observed (see Table 3). Thus, this approach may have boosted the less-is-more effects.

Another future direction, theoretically interesting, may involve a systematic manipulation of the familiarity, the complexity, and the degree of structuring of the decision problems presented. This may also include the degree of explicit specification of the goal to be attained (e.g., collecting funds vs. finding solutions to deal with the funding cut in our initial fundraising example). Perhaps a more generic specification of the goal may leave even more room for active structuring and ideation processes. It can be also worth exploring option generation scenarios in which novelty or originality of solutions are important (i.e., marketing, art and literature, scientific research). A deeper analysis of the impact of experience and knowledge of the domain on the option generation process may also be a direction to follow, as we have previously pointed out. A systematic manipulation of structural aspects of the problems will also allow moving from individual differences methods, which limit the possibility of making causal inferences, to experimental studies. In this regard, we consider the present research and its findings as a first significant step in a direction that may be followed both with individual-difference and with experimental methods, complemented with behavioral and neural process-tracing techniques.

### Conflict of interest statement

The authors declare that the research was conducted in the absence of any commercial or financial relationships that could be construed as a potential conflict of interest.

## Footnotes

^1^While the goal of idea generation tasks is to produce ideas not necessarily linked to potential courses of actions, option generation requires to produce (even abstract) courses of actions in response to a given decision problem, usually presented as a more constrained situation (for a detailed discussion see Kalis et al., 2013). Moreover, while idea generation task in creativity research generally focus on originality as one criterion for performance evaluation, this is not the case in option generation, where performance quality is generally evaluated in reference to utility of the generated courses of actions or to their effectiveness as solutions for the decision problem. However, both idea generation and option generation performance may depend, possibly to a different extent, on memory retrieval and idea generation processes.

^2^However, this model includes also some ideation components because it states that generating ideas from the retrieved information may require knowledge combination, new associations, or knowledge transfer.

^3^This conclusion is strengthened by the observation of similar cognitive difficulties in accessing alternative options reported in conceptually-related research on fault trees (e.g., Fischhoff et al., 1978; Russo and Kolzow, 1994), the predecisional focusing effect (Del Missier et al., 2007), and the part-set cuing effect in option generation and problem solving (Peynircioğlu, 1987; Del Missier and Terpini, 2009). Participants are also usually overconfident about the completeness of the option generation performance: They think they have generated a large fraction of the potential options, even if they have identified only a limited part of the existing possibilities (Mehle, 1982; Gettys et al., 1987).

^4^This time limit allows a proper assessment of generation performance, while avoiding extending too much the “empty” period that usually characterizes the final part of generation or free recall tasks (Harbison et al., 2009). Indeed, a pre-test and our own previous studies on option generation (e.g., Del Missier and Terpini, 2009) showed that a large majority of options are generated in the first 3–4 min. Given the repeated nature of the task, a prolonged experience of retrieval failures in the first scenario could have been potentially stressful and could have negatively affected performance in the subsequent option generation tasks.

^5^Participants reported to have more experience in the parking vs. the fund raising domain (*t* = 4.53, *df* = 140, *p* < 0.0001), and to have more experience in the energy saving vs. the parking domain (*t* = 4.93, *df* = 140, *p* < 0.0001) or the fund raising domain (*t* = 9.10, *df* = 140, *p* < 0.0001). The same pattern emerged for knowledge (parking vs. fund raising: *t* = 4.88, *df* = 140, *p* < 0.0001; energy saving vs. parking: *t* = 5.62, *df* = 140, *p* < 0.0001; energy saving vs. fund raising: *t* = 9.17, *df* = 140, *p* < 0.0001). Means on a 7-point scale for experience were as follows: 3.99 (parking), 3.18 (fund raising), 4.68 (energy saving). Means for knowledge were as follows: 4.11 (parking), 3.39 (fund raising), 4.73 (energy saving).

^6^Categories for the parking problem were: (1) use available parking space more efficiently, (2) increase available space for parking, (3) reduce demand for parking, (4) other (including Gettys et al.'s change parking priorities). Categories for the fund raising problem were: (1) fund raising from citizens, (2) fund raising from public institutions and organizations, (3) fund raising from companies, private sector, or nonpublic organizations, (4) investments, (5) other. Categories for the energy saving problem were: (1) lighting, (2) appliances, (3) electronic devices and tv, (4) hot water, (5) heating, (6) air conditioning and cooling, (7) structural interventions (e.g., insulation), (8) self-production, (9) other. We included self-production of energy to accommodate participants' answers, even if this is not considered as energy saving (but eventually money saving).

^7^In all the three problems investigated, participants who accessed more response categories also generated more options (parking: *r* = 0.52, fund raising: *r* = 0.45, energy saving: *r* = 0.66; *p* < 0.001; see also Del Missier and Terpini, 2009).

^8^In our study, as well as in previous ones (see e.g., Gettys et al., 1987; Klein et al., 1995), the “average individual” showed a rather partial generation performance if the whole option space is considered, both in terms of the number of options and of the number of categories accessed. As in previous studies, lower bound estimates of option spaces obtained from pooling responses of the (nominal) group of individual participants (see e.g., Gettys et al., 1987) indicated a much larger set of possibilities than the options generated by individual participants (parking = 122; fund raising = 75; energy saving = 121). For what concerns diversity, participants missed from 25% to more than 50% of the response categories, depending on the problem (see Supplementary Material Table 1A).
