# Supplementary material for: Option generation in decision making: ideation beyond memory retrieval
Source: Front Psychol. 2015 Jan 22;5:1584. doi: 10.3389/fpsyg.2014.01584 (PMC4302792; doi:10.3389/fpsyg.2014.01584)
Supplement: Supplementary file 1 [file DataSheet1.PDF]

APPENDIX – ADDITIONAL MATERIALS

Decision problems used in the option generation task

Parking problem.

A middle size city is currently experiencing parking problems in the city center, causing slowdowns, queues, and complaints among the population. The city administration decided to collect the citizens' suggestions to identify all the possible solutions to the parking problem in the city center. What would you suggest? Write down each possible solution as soon as it comes into your mind. You will have 6 minutes of time. Write as many possible solutions you can think of.

Fund raising problem

A nonprofit organization working for the defense of children's rights suffered a cut in public funding. To handle this situation, the association decided to collect members' and sympathizers' suggestions to identify all the possible ways to collect funds. What would you suggest? Write down each possible solution as soon as it comes into your mind. You will have 6 minutes of time. Write as many possible solutions you can think of.

Energy saving problem

Energy has become a fundamental issue in current society. There are various things that can be done to save energy in a house. If you were asked to say to other persons what they could do to reduce their domestic energy consumption, what would you suggest? Write down each possible solution as soon as it comes into your mind. You will have 6 minutes of time. Write as many possible solutions you can think of.

Item from the Applying Decision Rules subtest of the Adult Decision-Making Competence battery (Bruine de Bruin, Parker & Fischhoff, 2007)

Question 2:

Features

DVD		Picture Quality	Sound Quality	Programming Options	Reliability of Brand	Price
	A	2	5	5	5	$369
	B	5	4	4	5	$369
	C	5	3	2	5	$369
	D	3	5	2	2	$369
	E	4	4	4	5	$369

Sally first selects the DVD players with the best Sound Quality. From the selected DVD players, she then selects the best on Picture Quality. Then, if there is still more than one left to choose from, she selects the one best on Programming Options.

Which **one** of the presented DVD players would Sally prefer? ________________

Very Low	Low	Medium	High	Very High
1	2	3	4	5

Table 1A: Descriptive statistics for option generation fluency and diversity.

	Option Generation Fluency			Option Generation Diversity		
**Statistics**	*Parking*	*Fund raising*	*Energy saving*	*Parking*	*Fund raising*	*Energy saving*
Mean	5.79	5.76	7.09	2.46	2.05	4.30
95% CI	5.43 - 6.15	5.39 - 6.13	6.65 - 7.52	2.35 2.57	1.90 2.20	4.11 4.50
Median	5	6	7	3	2	4
Standard Deviation	2.16	2.23	2.61	0.66	0.90	1.19
Min-Max	2-13	2-13	2-15	1-4	1-4	2-7

Note: 95%CI = 95% confidence interval around the mean.

Table 2A: Pearson's Pairwise bivariate correlations between option generation performance (fluency and diversity), ADR performance, and their potential predictors.

	**Parking**		**Fund Raising**		**Energy Saving**		**Applying Decision Rules**
**Predictor**	*Fluency*	*Diversity*	*Fluency*	*Diversity*	*Fluency*	*Diversity*	*Score*
Cued Recall [a]	.11	-.02	.08	.05	**.19***	.11	**.31*****
Immediate	.07	-.05	.10	.05	.17	.08	**.27****
Delayed	.11	.00	.07	.01	**.19***	.11	**.33*****
Letter-Memory	.03	-.13	.06	.13	.10	.11	**.33*****
Stroop	.02	.01	-.06	-.03	-.05	-.15^	-.15^
Plus-minus	.04	.13	.07	.01	-.05	-.04	**-.17***
Category Fluency [a]	.14	.10	.07	.10	**.25****	.18*	.25**
Animal	.10	.02	.03	.05	.15^	.16^	**.23****
Fruits	.14^	.14^	.11	.12	**.27****	.15^	**.21***
Letter fluency [a]	.11	.05	.08	.06	.16^	.15	**.30*****
S	.13	.03	.09	.04	**.17***	**.18***	**.31*****
F	.10	.07	.10	.06	.16^	.11	**.26****
Raven's SPM	.03	-.08	-.04	.03	.01	.01	**.45*****
Cognitive Reflection	.08	-.05	.03	-.12	.14^	.14^	**.44*****
Alternative Uses [a]	**.44*****	**.26****	**.38*****	**.26****	**.50*****	**.28****	.09
Brick	**.40*****	**.21***	**.34***	**.22*****	**.44*****	**.18***	.05
Staple	**.38*****	**.24****	**.36*****	**.22***	**.45*****	**.32*****	.10
Tire	**.38*****	**.19***	**.29****	**.19***	**.35*****	**.19***	.05
Parking							
Knowledge	.08	.01	–	–	–	–	–
Experience	.16^	.01	–	–	–	–	–
Fund Raising							
Knowledge	–	–	.10	.09	–	–	–
Experience	–	–	.09	.05	–	–	–
Energy Saving							
Knowledge	–	–	–	–	**.26****	**.18***	–
Experience	–	–	–	–	**.25****	.13	–

Note. Two-tailed significance levels: *** p < .001; ** p < .01; * p < .05; ^ p < .10. Significant correlations are marked in bold.

[a] Compound variables for associative cued recall, category fluency, letter fluency, and alternative uses (ideation fluency) are derived by the unweighted average of the standardized scores of the respective tests.

Table 3A: Descriptive statistics for mean quality, max quality, and quality of choice.

**Mean Quality of Generated Options**	*Parking*	*Fund raising*	*Energy saving*
Mean	5.24	4.99	5.74
95% CI	5.08 – 5.40	4.88 – 5.09	5.66 – 5.83
Median	5.25	4.86	5.83
Standard Deviation	0.97	0.64	0.51
Min-Max	2.17 – 7	3.43 – 7	4.25 – 7
**Max Quality of Generated Options**	*Parking*	*Fund raising*	*Energy saving*
Mean	6.90	6.57	6.91
95% CI	6.82 – 6.98	6.44 - 6.70	6.84 - 6.98
Median	7	7	7
Standard Deviation	0.48	0.79	0.41
Min-Max	4 – 7	5 – 7	5 – 7
**Quality of the Chosen Option**	*Parking*	*Fund raising*	*Energy saving*
Mean	6.00	5.14	5.97
95% CI	5.71 – 6.29	4.91 – 5.38	5.76 – 6.18
Median	7	5	7
Standard Deviation	1.73	1.41	1.12
Min-Max	1 – 7	1 – 7	4 – 7

Note: 95%CI = 95% confidence interval around the mean. Scores are computed from rating scales ranging from 1 to 7.
